# 

**Published:** 1873-12

**Authors:** 


### Books and Pamphlets Received.

---

Treatise on the Disease of the Eye. Including the Anatomy of the Organ. By Dr. Carl Stellwag (von Carion). Translated from the Fourth German Edition and Edited by D. B. St. John Roosa, M. D.; Charles S. Bull, M. D., and Charles E. Hackley, M D. New York: Wm. Wood & Co., 1873. Buffalo: H. H. Otis.

A Manual of Midwifery. Including the Pathology of Pregnancy and the Puerpual State. By Dr. Karl Schroeder. Translated from the Third German Edition. By Charles H. Carter, B. A. M. D., B. S. Lord. New York: D. Appleton & Co., 1873. Buffalo: Martin Taylor.

A system of Midwifery, Including Diseases of Pregnancy and the Puerpual State. By William Leishman, M. D. Illustrated. Philadelphia: Henry C. Lea, 1873.

THE BUFFALO

### Medical and Surgical Journal

PUBLISHED MONTHLY,

Containing Original Articles, Reports of Medical Societies and Hospitals, Editorial, Reviews, Correspondence, Army News, etc., etc; including the usual variety of Medical Periodical Publications. Specimen copies sent on application.

Address Buffalo Medical & Surgical Journal, Buffalo, N. Y.

TERMS OF SUBSCRIPTION, - - $3.00 PER YEAR, IN ADVANCE.

